# Human Microfibrillar-Associated Protein 4 (MFAP4) Gene Promoter: A TATA-Less Promoter That Is Regulated by Retinol and Coenzyme Q10 in Human Fibroblast Cells

**DOI:** 10.3390/ijms21218392

**Published:** 2020-11-09

**Authors:** Ying-Ju Lin, An-Ni Chen, Xi Jiang Yin, Chunxiang Li, Chih-Chien Lin

**Affiliations:** 1 School of Chinese Medicine, China Medical University, Taichung 40447, Taiwan; yjlin.kath@gmail.com; 2 Genetic Center, Proteomics Core Laboratory, Department of Medical Research, China Medical University Hospital, Taichung 40447, Taiwan; 3 Department of Cosmetic Science, Providence University, Taichung 43301, Taiwan; g1030068@gm.pu.edu.tw; 4 Advanced Materials Technology Centre, Singapore Polytechnic, Singapore 139651, Singapore; YIN_Xi_Jiang@sp.edu.sg (X.J.Y.); LI_Chunxiang@sp.edu.sg (C.L.)

**Keywords:** elastic fibers, microfibrillar-associated protein 4 (MFAP4), TATA-less promoter, fibroblast cells

## Abstract

Elastic fibers are one of the major structural components of the extracellular matrix (ECM) in human connective tissues. Among these fibers, microfibrillar-associated protein 4 (MFAP4) is one of the most important microfibril-associated glycoproteins. MFAP4 has been found to bind with elastin microfibrils and interact directly with fibrillin-1, and then aid in elastic fiber formation. However, the regulations of the human *MFAP4* gene are not so clear. Therefore, in this study, we firstly aimed to analyze and identify the promoter region of the human *MFAP4* gene. The results indicate that the human *MFAP4* promoter is a TATA-less promoter with tissue- and species-specific properties. Moreover, the promoter can be up-regulated by retinol and coenzyme Q10 (coQ10) in Detroit 551 cells.

## 1. Introduction

Similar to the collagen fibers, elastic fibers are one of the major structural components of the extracellular matrix (ECM) in human connective tissues. Elastic fibers are responsible for repeated stretching in many tissues, such as in the skin, aorta, and lungs [1,2]. Elastic fibers have two main components: the elastin core, which is processed from tropoelastin, and the microfibrils, which are mainly composed of fibrillins [3]. Moreover, fibulins and microfibril-associated glycoproteins (MAGPs) are accessory proteins which may combine with elastin and/or microfibrils; some of them are also associated with elastic fiber maturation [4]. Therefore, elastic fibers are complex in their components and structural development. In addition to this, the dysfunction of elastin-related genes may cause severe conditions in humans. These include Marfan syndrome (MFS), a connective tissue disease that is caused by mutations in the fibrillin-1 (*FBN1*) gene [5,6].

Among the MAGPs, microfibrillar-associated protein 4 (MFAP4) is one of the most important. MFAP4 is recognized as the human homolog of MAGP-36, and it has been found to bind with elastin microfibrils [7]. An earlier study demonstrated that MFAP4 can interact directly with fibrillin-1 and promote elastic fiber formation, while MFAP4 expression also protects the skin from UVB-induced damage [8]. Evidently, MFAP4, as a ligand of microfibrils and tropoelastin regarding correct elastic fiber organization, may contribute to both the maturation and the maintenance of elastic fiber [9].

MFAP4 was also identified as a potential biomarker for cardiovascular disease [10], chronic obstructive pulmonary disease [11], and hepatic fibrosis [12]. It was also found that high levels of plasma MFAP4 correlated independently with diabetic neuropathy [13]. MFAP4-deficient mice display emphysema-like pathology and impaired formation of the neointimal hyperplasia [14]. Therefore, MFAP4 is very important due to it being associated with many human diseases. However, until now, the regulations of the human *MFAP4* gene have not been so clear, and there has not been any study focused on the analysis of the human *MFAP4* promoter region.

In eukaryotes, the genome has corresponding regulatory machinery containing various cis- and trans-elements. Among them, the cis-regulatory elements are mostly noncoding DNA sequences spread within the genome [15]. Moreover, the gene promoter can be defined as the combination of cis-regulatory elements that are essential for initiation of gene transcription. The promoter can also be known to enhance the frequency of initiation only when located near the transcriptional start site. Moreover, the promoter region mainly comprises the core promoter and proximal promoter elements; some of the known core promoter elements are the TATA box, the initiator element (Inr), the transcription factor IIB (TFIIB) recognition element (BRE), the downstream promoter element (DPE), and the motif ten element (MTE) [16].

In the presented study, we firstly aimed to analyze and identify the promoter region of the human *MFAP4* gene. The reporter gene systems, Enhanced Green Fluorescent Protein (EGFP) and Secreted Alkaline Phosphatase (SEAP), were utilized to analyze *MFAP4* promoter activity. In addition, the effects of retinol and coenzyme Q10 (coQ10) on the expression of the human *MFAP4* promoter region were also evaluated further.

## 2. Results

### 2.1. Prediction and Plasmid Construction of Human MFAP4 Promoter

**Figure 1:**
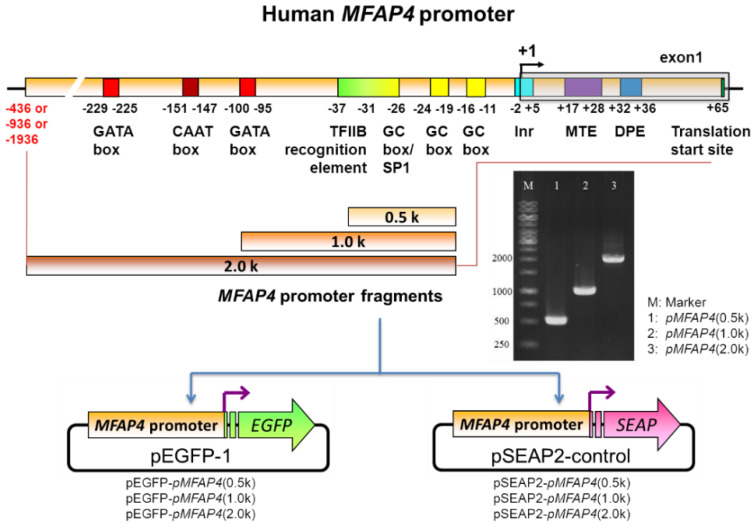
Illustration of the plasmid construction of human microfibrillar-associated protein 4 (*MFAP4*) promoter in this study.

**Figure 2:**
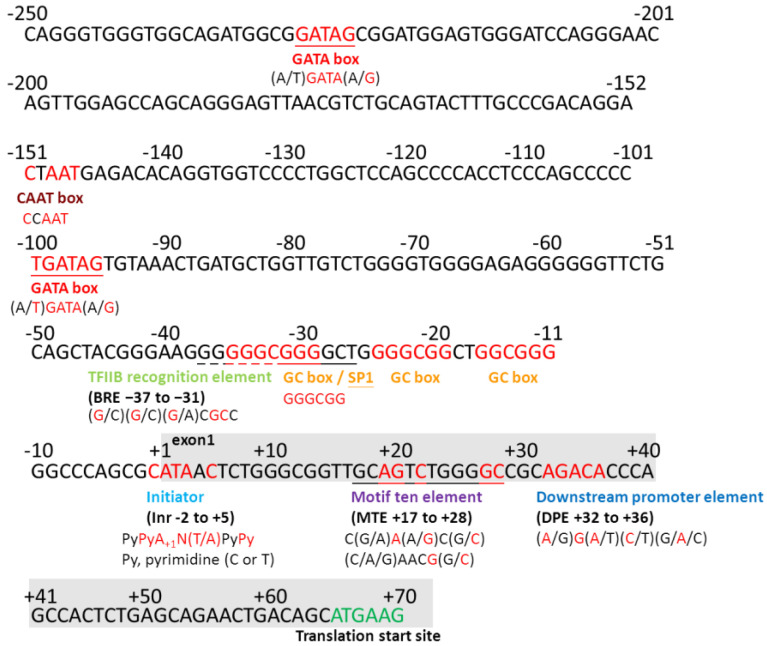
Prediction and analysis of the human *MFAP4* promoter. The presented region is from position +70 to −250. The corresponding elements and consensus sequences are shown at the bottom of the main sequence. Py: pyrimidine (C or T), N: any base.

The human (*Homo sapiens*) *MFAP4* gene is located on chromosome 17 at the region of 19,383,445 to 19,387,190 [17]. The gene derives two transcript variants; the mature mRNAs are 840 and 768 nucleotides (nts) in length, encoded with 279 and 255 amino acids of MFAP4 protein isoforms, and the transcription start sites for these two transcript variants are at the same position. If we set the transcription start site as +1, the chosen lengths for the testing of the predicted promoter regions were 0.5 k, 1.0 k, and 2.0 k base pairs (bps), as shown in Figure 1. Moreover, several potential functional elements were predicted, which are shown in Figure 1 and Figure 2.

To analyze the sequence of the promoter region in detail, we found that the human *MFAP4* promoter is a type of TATA-less promoter, which means that the proposed promoter region has no TATA-box located at the −25 positions for the eukaryotes (Figure 2). Furthermore, between the transcription and translation start sites, we can recognize several important motifs, including the initiator (Inr), motif ten element (MTE), and downstream promoter element (DPE). These core promoter motifs mainly drive and control the downstream gene sequence for a normal TATA-less promoter [18]. This is the main reason we chose the sequences from its translation start site to −436, −936, and −1936, which can generate 0.5k, 1.0k, and 2.0k bps promoter fragments (Figure 1). In addition, from sequences of +1 to −250, we also found many possible elements/boxes in the proximal promoter region, including the GC box, TFIIB recognition element, B recognition element (BRE), GATA box, and CAAT box (Figure 1 and Figure 2). Generally, there may also be some regulation elements such as enhancers or suppressors, mostly located within −2000 bps. Thus, three promoter DNA fragments with 0.5k, 1.0k, and 2.0k were firstly used to test the promoter activity in the present study.

Consequently, to investigate the functions of the predicted human *MFAP4* promoter regions, we used PCR to amplify these DNA fragments using designed primers (Table 1). The DNA electrophoresis for PCR products is shown in Figure 1. From lane 1 to lane 3, we can observe the amplified DNA fragments of the *MFAP4* promoter with 0.5k to 2.0k. Then, the purified DNA fragments were cloned into vectors pEGFP-1 and pSEAP2-control to generate plasmids pEGFP-*pMFAP4*(0.5k), pEGFP-*pMFAP4*(1.0k), pEGFP-*pMFAP4*(2.0k), pSEAP2-*pMFAP4*(0.5k), pSEAP2-*pMFAP4*(1.0k), and pSEAP2-*pMFAP4*(2.0k), respectively (Figure 1). The expressed reporter EGFP in the cell normally stays at the cytosols and can be rapidly observed by a fluorescence microscope or measured by an enzyme-linked immunosorbent assay (ELISA) reader. By contrast, reporter SEAP is a kind of secreted enzyme that can remove the phosphate moiety from the substrate. It mimics the secretory process of extracellular matrix proteins, such as elastic fiber-related proteins, including MFAP4.

### 2.2. Expression of pEGFP-pMFAP4 Plasmids in Human Fibroblast Cells

**Figure 3:**
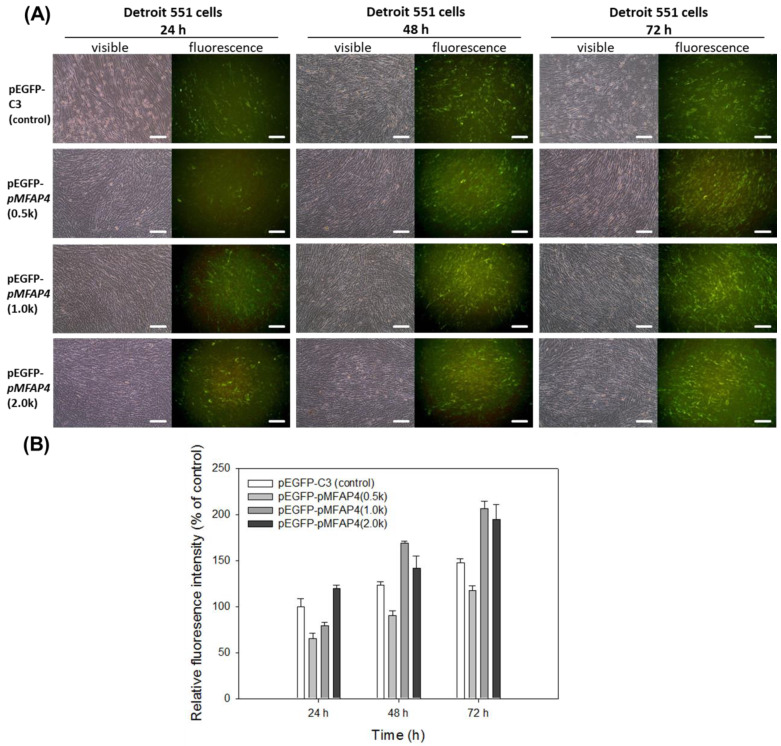
The expression of pEGFP-*pMFAP4* plasmids in human fibroblast Detroit 551 cells. (**A**) Fluorescence images. Scale bar = 100 μm. (**B**) Relative fluorescence intensity. Data are presented as means ± SE.

To confirm the activities of the proposed human *MFAP4* promoters in human fibroblast cells, we transfected the pEGFP-*pMFAP4* plasmids into Detroit 551 cells. At 24 to 72 h post transfection, the fluorescent images and intensities were recorded; the results are shown in Figure 3. Every analyzed plasmid was measured by the co-transfection of control plasmid (pDsRed) at the ratio of 10:1, and then the results were normalized to transfection efficiencies. All the transfected plasmids, including pEGFP-*pMFAP4*(0.5k), pEGFP-*pMFAP4*(1.0k), and pEGFP-*pMFAP4*(2.0k), were able to drive EGFP expression in Detroit 551 cells at every time point (Figure 3A). Therefore, all the proposed human MFAP4 promoters are functional in human fibroblasts. In addition to this, compared with the control plasmid pEGFP-1, pEGFP-*pMFAP4*(1.0k) and pEGFP-*pMFAP4*(2.0k) expressed relatively higher fluorescence intensities at 48 and 72 h. Moreover, the fluorescence intensity of pEGFP-*pMFAP4*(0.5k)-transfected Detroit 551 cells was lower than that of the other groups at every time point.

### 2.3. Expression of pEGFP-pMFAP4 Plasmids in Mouse Fibroblasts and in Human Melanoma and Mouse Melanoma Cells

**Figure 4:**
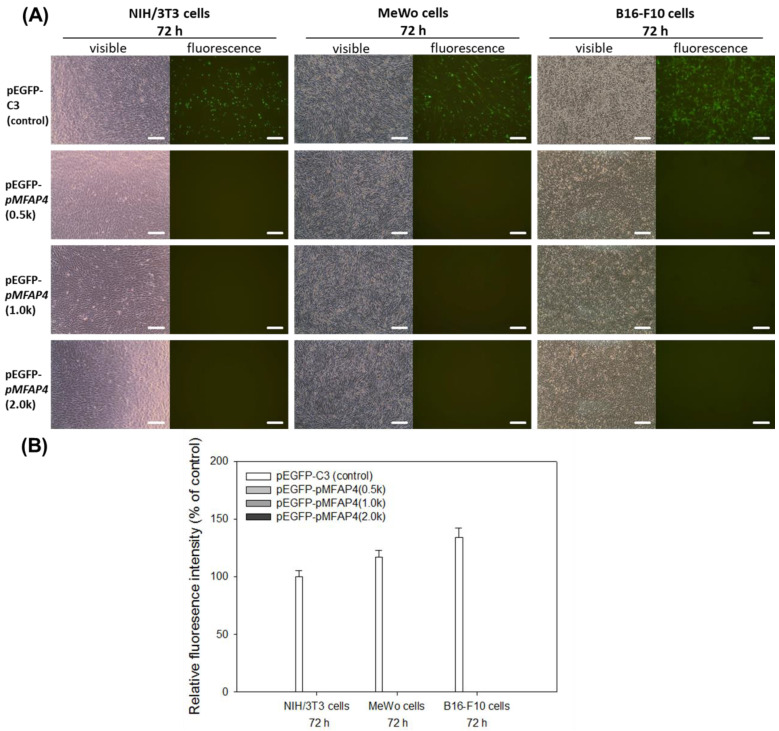
The expression of pEGFP-*pMFAP4* plasmids in mouse fibroblast NIH/3T3 cells, human melanoma MeWo cells, and mouse melanoma B16-F10 cells. (**A**) Fluorescence images. Scale bar = 100 μm. (**B**) Relative fluorescence intensity. Data are presented as means ± SE.

To further evaluate the activity of human *MFAP4* promoter, we transfected the plasmids into mouse fibroblasts (NIH/3T3 cells), human melanoma cells (MeWo cells), and mouse melanoma cells (B16-F10 cells). The results are shown in Figure 4. Our results indicated that the human *MFAP4* promoter cannot drive reporter gene expression in these other three cell lines at 72 h, unlike Detroit 551 cells, including all the tested promoter regions. Furthermore, the transfected plasmids of human *MFAP4* promoter in three cell lines were not only silent at 72 h, but also at every measured time points from 24 to 48 h (data not shown). Although the control plasmid pEGFP-1 can still express EGFP in NIH/3T3, MeWo, and B16-F10 cells, EGFP intensity was not observed in the other pEGFP-*pMFAP4*(0.5k), pEGFP-*pMFAP4*(1.0k), or pEGFP-*pMFAP4*(2.0k) transfected cells, even in the human cell line, MeWo cells.

### 2.4. The Alignment of the Human MFAP4 Promoter Region with Those of Other Species

**Figure 5:**
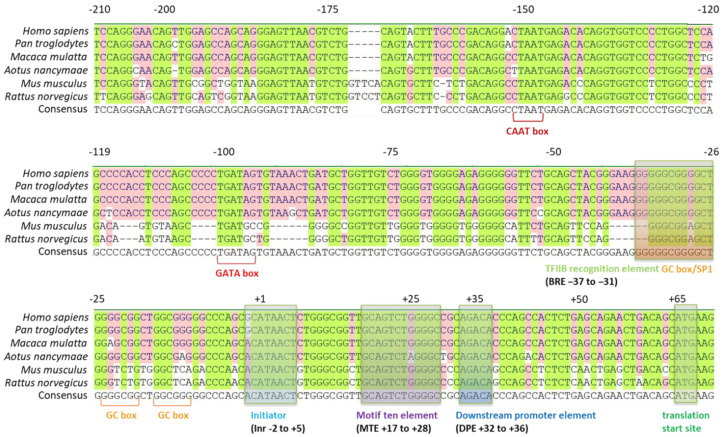
The alignment of the human *MFAP4* promoter region with those of other species.

The human *MFAP4* promoter revealed a species-specific feature that is activated only in human cells. Hence, we compared the proposed *MFAP4* core promoter region of humans (*Homo sapiens*) with those of chimpanzees (*Pan troglodytes*), Ma’s night monkeys (*Aotus nancymaae*), rhesus monkeys (*Macaca mulatta*), mice (*Mus musculus*), and rats (*Rattus norvegicus*) to further verify the features of the *MFAP4* core promoter region. The result is shown in Figure 5. Most of the core promoter moieties are highly conserved in the tested species, including Inr, MTE, and DPE. However, in the region of −1 to −210, including the BRE, TFIIB recognition element, and other parts, the sequences can be divided into two types. The same as the human sequence, the chimpanzee, Ma’s night monkey, and rhesus monkey (all primates) sequences can be classified in one group, then the mouse and rat sequences form another group (Figure 5). Although the similarity of these species is high, the difference in the *MFAP4* promoter core region between a human and a mouse/rat is still obvious.

### 2.5. Effect of Retinol and coQ10 on the Expression of Human MFAP4 Promoter in Detroit 551 Cells

**Figure 6:**
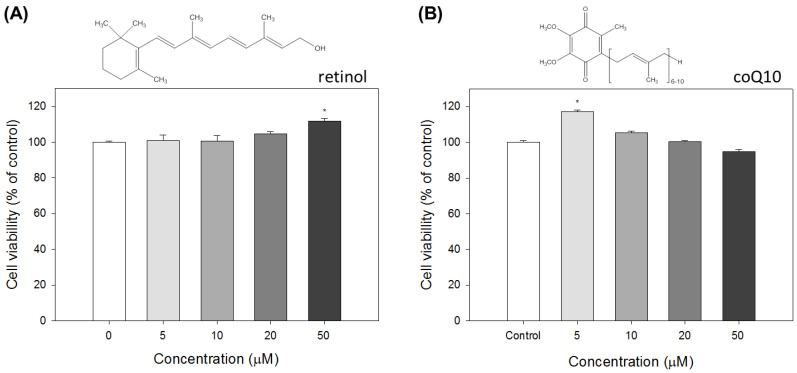
Cytotoxicity of (**A**) retinol and (**B**) coenzyme Q10 (coQ10) in human fibroblast Detroit 551 cells. Data are presented as means ± SE; * indicates a *p* value of less than 0.05 when compared with the control.

To test the effects of retinol and coenzyme Q10 on the expression of the human *MFAP4* promoter in fibroblast cells, we firstly confirmed the cytotoxicity of each compound using the standard MTT assay. Subsequently, the effect of these compounds on promoter activities could be measured in the proper concentrations. The structures of retinol and coQ10 are presented in Figure 6. The results indicated that the viability of Detroit 551 cells is slightly increased with growing retinol concentrations: at concentrations of retinol above 50 μM, the cell viability increased to approximately 110% that of the control (Figure 6A). Thus, to avoid the influence of increasing cell proliferation on promoter activity measurements, we used 20 μM retinol for the following experiments. By contrast, treatment of coQ10 at concentrations higher than 5 μM had no obvious effects on the viability of Detroit 551 cells. Moreover, at 50 μM, coQ10 slightly decreased the viability of Detroit 551 cells. Therefore, 20 μM was the selected concentration of coQ10 for future experiments.

**Figure 7:**
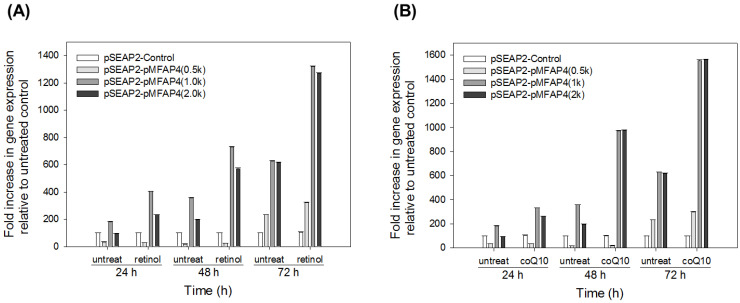
Effect of (**A**) retinol and (**B**) coQ10 on the expression of the human *MFAP4* promoter in Detroit 551 cells. Data are presented as means ± SE.

Our earlier study proved that retinol and coQ10 can enhance the expression of tropoelastin and fibrillin-1 in human fibroblasts [19]. Therefore, we investigated the effects of retinol and coQ10 on human *MFAP4* promoter activity in Detroit 551 cells. The plasmids pSEAP2-*pMFAP4*(0.5k), pSEAP2-*pMFAP4*(1.0k), and pSEAP2-*pMFAP4*(2.0k) were transfected into Detroit 551 cells, then the effects of retinol and coQ10 on human *MFAP4* promoters were measured at 24, 48, and 72 h. The results are shown in Figure 7. When compared with each untreated group (control), we can see that both retinol and coQ10 evidently up-regulated the expression of human *MFAP4* promoter activities in the Detroit 551 cells. The plasmids pSEAP2-*pMFAP4*(1.0k) and pSEAP2-*pMFAP4*(2.0k) were enhanced in terms of expression by both retinol and coQ10 at every tested time point (Figure 7A,B), the expression being around 1.8 to 2.2 times that of the untreated control. The increase in the expression of *MFAP4* promoters (both 1.0k and 2.0k) due to coQ10 was higher than that due to retinol at 48 h (Figure 7B). Moreover, for pSEAP2-*pMFAP4*(0.5k), the effects of retinol and coQ10 could only be detected at the 72 h time point, the increase being approximately 40% to 50% compared to the untreated control. Thus, the results indicated that retinol and coQ10 are evidently effective for the expression of human *MFAP4* promoter.

## 3. Discussion

In humans, many genes are only expressed in a few cellular types to restrict the specific protein expression to a precise position. These include the promoters of fibroblast growth factor type 1 (FGF1), fibroblast growth factor 21 (FGF21), and collagen α1(XI) (COL11A1) in fibroblasts [20,21,22], due to the fact that fibroblasts are the cells majorly expressing ECM in the human body. Therefore, combining our results (Figure 3 and Figure 4), we can suppose that the human *MFAP4* promoter is a kind of tissue-specific promoter that is functional only in the elastin-producing cells (such as fibroblasts), but not in the melanocytes. Moreover, to further confirm this finding, we have transfected the pSEAP2-*pMFAP4*(0.5k), pSEAP2-*pMFAP4*(1.0k), and pSEAP2-*pMFAP4*(2.0k) plasmids into another human fibroblast from normal lung tissue, MRC-5 cells (BCRC 60023), and the results revealed that human *MFAP4* promoter is also functional in MRC-5 cells, similar to that in Detroit 551 cells (data not shown). The results are summarized in Table 2.

Additionally, it is a species-specific promoter that is activated only in human cells. In the results of alignment of the human *MFAP4* promoter region with those of other species, although the similarity of these species is high, we can also find some noticeable differences between humans and mice/rats in the *MFAP4* promoter core region. This may be one of the key reasons the human *MFAP4* promoter cannot activate in mouse cell lines, which reveals the species-specific characteristic. However, on the other hand, the *MFAP4* promoter is also a kind of tissue-specific promoter, and it is often correlated with the regulatory components within the cell, such as transcription factors. The general transcription factor, TFIID, is a large and multiple assembly that serves as a general transcription factor for gene transcription initiation by RNA polymerase II in the eukaryotes. In addition, TFIID is involved in the recognition of the core promoter sequences and adjacent chromatin marks, and it also interacted with some gene-specific regulators [23]. TFIID is responsible for the recognition of mostly known core promoter region, but that is an exception of the BRE, which is targeted by another transfection factor TFIIB. In our results, there is a predicated BRE sequence found in the human *MFAP4* promoter between the positions of −31 to −37 (Figure 2 and Figure 5). Therefore, it might be the reason that the human *MFAP4* promoter can be only activated in some elastin-producing cells.

Retinol, a topical vitamin A derivative, has been used to reverse the visual presence changes associated with premature aging and improve the looks of skin [24]. Furthermore, coQ10 is an endogenous lipophilic quinone that exists in every biological membrane with the antioxidant and bioenergetic properties, and those are all related to the aging process. In addition, coQ10 synthesis is known to decrease with age in different tissues, including skin [25]. As a result, further investigating retinol and coQ10′s enhancing effects on the expression of elastic fiber components (including MFAP4) may be a good strategy for people to discover new potential materials that can be used in anti-aging cosmetics.

## 4. Materials and Methods

### 4.1. Materials

Coenzyme Q10 (coQ10) was obtained from Nisshin Pharma Inc. (Tokyo, Japan). Magnesium chloride (MgCl_2_), L-homoarginine, diethanolamine and para-nitrophenyl phosphate (pNPP), ethidium bromide, dithiothreitol (DTT), agarose (for DNA electrophoresis), retinol, and other experimental chemicals were purchased from Sigma-Aldrich (St. Louis, MO, USA). Minimum essential medium alpha (α-MEM), minimum essential medium (MEM), Dulbecco’s modified Eagle medium (DMEM), L-glutamine, penicillin–streptomycin, deoxynucleotide triphosphate (dNTP), oligo(dT), *Pfu* DNA polymerase, and Moloney Murine Leukemia virus (M-MLV) reverse transcriptase were purchased from Gibco BRL/Invitrogen (Carlsbad, CA, USA). Restriction enzymes were purchased from New England BioLabs (Beverly, MA, USA). Fetal bovine serum (FBS) was purchased from HyClone (Logan, UT, USA). 3-(4,5-Dimethylthiazol-2-yl)-2,5-diphenyl tetrazolium bromide (MTT) was obtained from Affymetrix/USB (Cleveland, OH, USA). Xfect^TM^ transfection reagent was purchased from Clontech Laboratories, Inc. (Mountain View, CA, USA). The deionized distilled water (ddH_2_O) used for all solutions was purified using a Milli-Q system (Millipore, Bedford, MA, USA).

### 4.2. Plasmid Construction and Polymerase Chain Reaction (PCR) Experiments

The genomic DNA from Detroit 551 human normal fibroblast cells was extracted using a FavorPrep^TM^ Blood/Cultured Cell Genomic DNA Extraction Mini Kit (Favorgen, PingTung, Taiwan, ROC). DNA fragments of the human *MFAP4* promoter with different lengths (Figure 1) were amplified from the extracted genomic DNA using the *Pfu* DNA polymerase with our designed primers (Table 1). The amplified promoter DNA fragments were then cloned into pEGFP-1 and pSEAP2-Control vectors (Clontech, Mountain View, CA, USA) with *Xho*I and *Eco*RI restriction sites to generate the plasmids of pEGFP-*pMFAP4*(0.5k), pEGFP-*pMFAP4*(1.0k), pEGFP-*pMFAP4*(2.0k), pSEAP2-*pMFAP4*(0.5k), pSEAP2-*pMFAP4*(1.0k), and pSEAP2-*pMFAP4*(2.0k), respectively (Figure 1). The PCR reaction in this study was achieved using 1 μg plasmid DNA, 1.5 mM MgCl_2_, 0.2 mM dNTP, 2.5 units of *Taq* DNA polymerase, and 0.1 μM of each of the primers (Table 1). The annealing temperature was set to 55 °C and the amplifying process consisted of 35 cycles. The amplified DNA products were separated in 2% agarose gel in Tris-borate-EDTA (TBE) buffer and then stained with ethidium bromide for observation.

### 4.3. Cell Lines and Culture Conditions

Detroit 551 cells (BCRC 60118, human normal fibroblast cells), MeWo cells (BCRC 60540, human melanoma cells), NIH/3T3 cells (BCRC 60008, mouse fibroblast cells), and B16-F10 cells (BCRC 60031, mouse melanoma cells) were all obtained from the Bioresource Collection and Research Center (BCRC, Hsinchu, Taiwan, ROC). Detroit 551 cells were cultured in α-MEM, Detroit 551 cells were cultured in α-MEM, MeWo and NIH/3T3 cells were cultured in MEM, and the B16-F10 cells were cultured in DMEM. All media were supplemented with 10% FBS and 1% penicillin–streptomycin (100 U/mL penicillin and 100 μg/mL streptomycin). The cells were maintained in a humidified incubator at 37 °C with 5% CO_2_ and sub-cultured every 3–4 days to maintain logarithmic growth. All the cell lines were grown for 24 h before transfection or treatment.

### 4.4. Cell Transfection

The prepared cells were cultured in 24-well plates with 8 × 10^4^ cells/well, and the purified plasmid DNAs were transfected using Xfect^TM^ transfection reagent into cells at a concentration of 1 μg/well, according to the manufacturer’s protocol. After 4 h of transfection (25 °C), the transfection process was completed by the replacing of fresh medium. For transfection efficiency, all analyzed plasmids were measured by the co-transfection of control plasmid (pDsRed) at a ratio of 10:1.

### 4.5. Fluorescent Protein Intensity Measurement

The transfected cells were placed in 24-well plates with around 1 × 10^5^ cells/well. Then, the medium was replaced with phosphate-buffered saline (PBS) in each plate. Analyses of the fluorescence and its intensity were performed using an inverted fluorescence microscope (Olympus CKX41SF, Olympus Corporation, Japan) and an ELISA microplate reader (QuantTM, Bio-Tek, GA, USA), respectively. In the fluorescence microscope and ELISA microplate reader, the measurements were all established by excitation/emission at 488/507 nm for EGFP. The fluorescence microscope is mainly used to record the images, and the ELISA microplate reader can analyze the fluorescent intensity. The measured intensity percentages are expressed as the relative fluorescent intensity [26].

### 4.6. The Alignment of the Human MFAP4 Promoter Region with Those of Other Species

The *MFAP4* promoter sequences of humans (*Homo sapiens*) and other species used in this study were all obtained from the Genome or Nucleotide database of the National Center for Biotechnology Information (NCBI). The other species were chimpanzee (*Pan troglodytes*), Ma’s night monkey (*Aotus nancymaae*), rhesus monkey (*Macaca mulatta*), mouse (*Mus musculus*), and rat (*Rattus norvegicus*). Alignment of the human *MFAP4* promoter region to those of other species was performed using the Basic Local Alignment Search Tool (BLAST) from NCBI.

### 4.7. Cell Viability Assay (MTT Assay)

The Detroit 551 cells were seeded in 96-well plates at a density of 8 × 10^3^ cells/well. After 24 h of incubation, the cells were then treated with different concentrations of compounds for 24 h. Next, 100 μL MTT solution at a concentration of 0.5 mg/mL was added to the cells, which were incubated at 37 °C for 4 h and then washed twice with PBS. Last of all, the prepared cells were lysed with 100 μL DMSO, and the absorbance was measured spectrophotometrically at 540 nm using an ELISA reader [27].

### 4.8. Compound Treatments for Promoter Activity Analysis

Detroit 551 cells were cultured in 24-well plates with 8 × 10^4^ cells/well for 24 h, and the cells were subsequently transfected with 1 μg/well plasmids using Xfect^TM^ transfection reagent. At 24 h post transfection, cells were treated with or without (untreated group) different concentrations of retinal or coQ10 for another 24 h. The cell culture supernatants were collected at different times (24 to 72 h) and then analyzed for SEAP activity.

### 4.9. SEAP Activity Assay

For the SEAP activity assay, the cell culture supernatants were placed at 65 °C for 10 min to eliminate endogenous alkaline phosphatase activities, then the samples were centrifuged at 14,000× *g* for 1 min. We added 300 μL samples with an equal volume of 2X SEAP reaction buffer (2 M diethanolamine, 1 mM MgCl_2_, 20 mM L-homoarginine, pH 9.8) to each well with the enzyme (phosphatase) substrate (20 mM pNPP). The pNPP is dephosphorylated by SEAP to generate p-nitrophenol; the spectrometric analysis can perform at 405 nm. The kinetic assay was performed by reading the absorbance at 405 nm at regular intervals over a 10 min period using an ELISA microplate reader [28].

### 4.10. Statistical Analysis

The quantitative data for this study were analyzed using Student’s *t*-tests and presented as the mean ± SE. The experiments were performed independently three times.

## 5. Conclusions

In summary, we analyzed the promoter region of the human *MFAP4* gene. The effects of retinol and coQ10 on the expression of the human *MFAP4* promoter region were also evaluated. The results indicated that the promoter is functional and is a tissue- and species-specific promoter. In addition, this TATA-less promoter can be up-regulated by retinol and coQ10 in Detroit 551 cells. The results may also emphasize the importance of MFAP4 in human tissue. Moreover, these findings may also aid in the discovery of potential cosmetic ingredients in the future.

## Figures and Tables

**Table 1:** The primers used in the study.

Primer name	Sequence
hMFAP4-P-436-XhoI-F	5′-g**CTCGAG**CCCAGAAGCCAGGCATATGA-3′
hMFAP4-P-936-XhoI-F	5′-g**CTCGAG**GAGGCAATAGGGAGCCATGA-3′
hMFAP4-P-1936-XhoI-F	5′-g**CTCGAG**TTTACAGAGGAGGAAACCAAGG-3′
hMFAP4-P+64-EcoRI-R	5′-g**GAATTC**GCTGTCAGTTCTGCTCAGAGT-3′

The sequences with bold letters represent restriction sites.

**Table 2:** The comparison of hMFAP4 promoter expression in the tested cell lines.

Cell Lines	Species	Features (Tissue/Type)	Elastin Production/*hMFAP4* Promoter Activity
Detroit 551 (BCRC 60118)	Human	Skin/normal/fibroblast	Y/Y
MeWo (BCRC 60540)	Human	Skin/melanoma	N/N
NIH/3T3 (BCRC 60008)	Mouse	Embryo/fibroblast	Y/N
B16-F10 (BCRC 60031)	Mouse	Skin/melanoma	N/N
MRC-5 (BCRC 60023)	Human	Lung/normal/fibroblast	Y/Y

Y represents yes, N represents no.

## References

[1] Etich J., Koch M., Wagener R., Zaucke F., Fabri M., Brachvogel B. (2019). Gene Expression Profiling of the Extracellular Matrix Signature in Macrophages of Different Activation Status: Relevance for Skin Wound Healing. Int. J. Mol. Sci..

[2] Eckersley A., Mellody K.T., Pilkington S., Griffiths C.E.M., Watson R.E.B., O’Cualain R., Baldock C., Knight D., Sherratt M.J. (2018). Structural and compositional diversity of fibrillin microfibrils in human tissues. J. Biol. Chem..

[3] Philips N., Chalensouk-Khaosaat J., Gonzalez S. (2018). Simulation of the Elastin and Fibrillin in Non-Irradiated or UVA Radiated Fibroblasts, and Direct Inhibition of Elastase or Matrix Metalloptoteinases Activity by Nicotinamide or Its Derivatives. J. Cosmet. Sci..

[4] Combs M.D., Knutsen R.H., Broekelmann T.J., Toennies H.M., Brett T.J., Miller C.A., Kober D.L., Craft C.S., Atkinson J.J., Shipley J.M. (2013). Microfibril-associated glycoprotein 2 (MAGP2) loss of function has pleiotropic effects in vivo. J. Biol. Chem..

[5] Bonetti M.I. (2009). Microfibrils: A cornerstone of extracellular matrix and a key to understand Marfan syndrome. Ital. J. Anat. Embryol. Arch. Ital. Anat. Embriol..

[6] Schrenk S., Cenzi C., Bertalot T., Conconi M.T., Di Liddo R. (2018). Structural and functional failure of fibrillin1 in human diseases (Review). Int. J. Mol. Med..

[7] Hirano E., Fujimoto N., Tajima S., Akiyama M., Ishibashi A., Kobayashi R., Okamoto K. (2002). Expression of 36-kDa microfibril-associated glycoprotein (MAGP-36) in human keratinocytes and its localization in skin. J. Dermatol. Sci..

[8] Kasamatsu S., Hachiya A., Fujimura T., Sriwiriyanont P., Haketa K., Visscher M.O., Kitzmiller W.J., Bello A., Kitahara T., Kobinger G.P. (2011). Essential role of microfibrillar-associated protein 4 in human cutaneous homeostasis and in its photoprotection. Sci. Rep..

[9] Pilecki B., Holm A.T., Schlosser A., Moeller J.B., Wohl A.P., Zuk A.V., Heumuller S.E., Wallis R., Moestrup S.K., Sengle G. (2016). Characterization of Microfibrillar-associated Protein 4 (MFAP4) as a Tropoelastin- and Fibrillin-binding Protein Involved in Elastic Fiber Formation. J. Biol. Chem..

[10] Wulf-Johansson H., Lock Johansson S., Schlosser A., Trommelholt Holm A., Rasmussen L.M., Mickley H., Diederichsen A.C., Munkholm H., Poulsen T.S., Tornoe I. (2013). Localization of microfibrillar-associated protein 4 (MFAP4) in human tissues: Clinical evaluation of serum MFAP4 and its association with various cardiovascular conditions. PLoS ONE.

[11] Johansson S.L., Roberts N.B., Schlosser A., Andersen C.B., Carlsen J., Wulf-Johansson H., Saekmose S.G., Titlestad I.L., Tornoe I., Miller B. (2014). Microfibrillar-associated protein 4: A potential biomarker of chronic obstructive pulmonary disease. Respir. Med..

[12] Molleken C., Poschmann G., Bonella F., Costabel U., Sitek B., Stuhler K., Meyer H.E., Schmiegel W.H., Marcussen N., Helmer M. (2016). MFAP4: A candidate biomarker for hepatic and pulmonary fibrosis?. Sarcoidosis Vasc. Diffus. Lung Dis. Off. J. Wasog.

[13] Blindbaek S.L., Schlosser A., Green A., Holmskov U., Sorensen G.L., Grauslund J. (2017). Association between microfibrillar-associated protein 4 (MFAP4) and micro- and macrovascular complications in long-term type 1 diabetes mellitus. Acta Diabetol..

[14] Zhu S., Ye L., Bennett S., Xu H., He D., Xu J. (2020). Molecular structure and function of microfibrillar-associated proteins in skeletal and metabolic disorders and cancers. J. Cell. Physiol..

[15] Consortium E.P. (2012). An integrated encyclopedia of DNA elements in the human genome. Nature.

[16] Bylino O.V., Ibragimov A.N., Shidlovskii Y.V. (2020). Evolution of Regulated Transcription. Cells.

[17] Zhao Z., Lee C.C., Jiralerspong S., Juyal R.C., Lu F., Baldini A., Greenberg F., Caskey C.T., Patel P.I. (1995). The gene for a human microfibril-associated glycoprotein is commonly deleted in Smith-Magenis syndrome patients. Hum. Mol. Genet..

[18] Vo Ngoc L., Kassavetis G.A., Kadonaga J.T. (2019). The RNA Polymerase II Core Promoter in Drosophila. Genetics.

[19] Lin C.C., Yang C.H., Kuo W.T., Chen C.Y. (2015). Evaluation of Anti-aging Compounds Using the Promoters of Elastin and Fibrillin-1 Genes Combined with a Secreted Alkaline Phosphatase Reporter in Normal Human Fibroblasts. Curr. Pharm. Biotechnol..

[20] Liu S.W., Hsu C.H., Chen M.R., Chiu I.M., Lin K.M. (2019). A Tri-fusion Reporter Mouse Reveals Tissue-Specific FGF1B Promoter Activity in vivo. Sci. Rep..

[21] Nygaard E.B., Moller C.L., Kievit P., Grove K.L., Andersen B. (2014). Increased fibroblast growth factor 21 expression in high-fat diet-sensitive non-human primates (Macaca mulatta). Int. J. Obes..

[22] Kinoshita A., Greenwel P., Tanaka S., Di Liberto M., Yoshioka H., Ramirez F. (1997). A transcription activator with restricted tissue distribution regulates cell-specific expression of alpha1(XI) collagen. J. Biol. Chem..

[23] Patel A.B., Greber B.J., Nogales E. (2020). Recent insights into the structure of TFIID, its assembly, and its binding to core promoter. Curr. Opin. Struct. Biol..

[24] Bjerke D.L., Li R., Price J.M., Dobson R.L.M., Rodrigues M., Tey C., Vires L., Adams R.L., Sherrill J.D., Styczynski P.B. (2020). The vitamin A ester retinyl propionate has a unique metabolic profile and higher retinoid-related bioactivity over retinol and retinyl palmitate in human skin models. Exp. Derm..

[25] Marcheggiani F., Cirilli I., Orlando P., Silvestri S., Vogelsang A., Knott A., Blatt T., Weise J.M., Tiano L. (2019). Modulation of Coenzyme Q10 content and oxidative status in human dermal fibroblasts using HMG-CoA reductase inhibitor over a broad range of concentrations. From mitohormesis to mitochondrial dysfunction and accelerated aging. Aging.

[26] Lin C.C., Yang C.H., Lin Y.J., Chiu Y.W., Chen C.Y. (2015). Establishment of a melanogenesis regulation assay system using a fluorescent protein reporter combined with the promoters for the melanogenesis-related genes in human melanoma cells. Enzym. Microb. Technol..

[27] Al-Hasawi N.A., Amine S.A., Novotny L. (2018). The In Vitro Anti-Proliferative Interaction of Flavonoid Quercetin and Toxic Metal Cadmium in the 1321N1 Human Astrocytoma Cell Line. Sci. Pharm..

[28] Lee S.M., Chiang S.H., Wang H.Y., Wu P.S., Lin C.C. (2015). Curcumin enhances the production of major structural components of elastic fibers, elastin, and fibrillin-1, in normal human fibroblast cells. Biosci. Biotechnol. Biochem..

